# Estimation of the transition rates in the illness-death model for chronic diseases from aggregated current status data: a feasibility and simulation study

**DOI:** 10.3389/fepid.2025.1691459

**Published:** 2025-12-19

**Authors:** Ralph Brinks, Maryam Mohammadi Saem, Sabrina Voß

**Affiliations:** Chair for Medical Biometry and Epidemiology, Faculty of Health/School of Medicine, Witten/Herdecke University, Witten, Germany

**Keywords:** prevalence, diabetes, epidemiology, study design, estimation, differential equations

## Abstract

Recently, it has been shown that the transition rates of the illness-death model (IDM) for chronic conditions are related to the age-specific prevalence by a partial differential equation (PDE). Given mortality, the PDE could be used to estimate incidence rates from cross-sectional data. The aim of this article is to extend the IDM and introduce a novel method to estimate the age-specific incidence rate together with the two mortality rates from aggregated current status (ACS) data. By ACS data we mean counts of people in the four states of the extended IDM at different points in time. ACS data stem from epidemiological studies where only current disease status and vital status data need to be collected without following-up people (as, for example, in cohort studies). To demonstrate feasibility of the method, we use a simulation study from the context of diabetes in Germany. Two estimation methods are introduced, a least squares estimator and a maximum likelihood estimator. We find a good agreement between the estimates and the input parameters used to set up the simulation.

## Introduction

**Figure 1:**
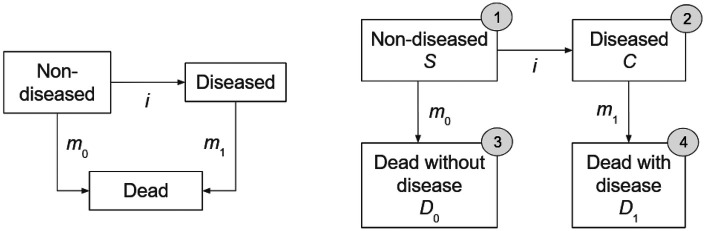
Two illness-death models (IDMs) for chronic diseases with transition rates *i*, *m*_0_, and *m*_1_ between the states. The conventional IDM (left) consists of the three states *Non-diseased*, *Diseased* and *Dead*. The extended IDM (right) distinguishes between *Dead without disease* and *Dead with disease*.

It could recently be shown that the transition rates *i*, *m*_0_, and *m*_1_ in the illness-death model (IDM, see left part of Figure 1) for chronic diseases are linked to the age-specific prevalence of the disease via a differential equation (DE) (1). Given information about mortality rates, the DE could be used to estimate the incidence from the prevalence (2) or to project future case-numbers of chronic diseases based on assumptions about the future transition rates (“scenarios”) (3). In the case that prevalence and incidence of the chronic condition are given, measures of excess mortality could be derived from the DE (4), although diagnostic accuracy turned out to be very important (5).

The aim of this article is to extend the IDM by an additional state and derive a system of DEs to estimate all three rates, *i*, *m*_0_, and *m*_1_ from aggregated current status (ACS) data. ACS data is closely connected to a state model (such as the IDM) and the information that we have available is the number of people in each of the states of the associated state model at specific points in time. The points in time are called monitoring times (6), and at each of the monitoring times, we only have the total counts of people in the health states. This is advantageous in settings with high data protection standards like, for example, in health insurance claims data [see e.g., (2)].

## Methods

### Extended IDM and system of DEs

We extend the conventional IDM as depicted in the left part of Figure 1 by separating the state *Dead* in Figure 1 into two different (disjunct) states, *Dead without disease* and *Dead with disease*. This means, we differentiate whether a deceased subject has contracted the disease before death or not. Hence, each subject of the population under consideration is assigned to exactly one the four states shown in the right part of Figure 1: *Non-diseased* (state 1 in Figure 1), *Diseased* (2), *Dead without disease* (3) and *Dead with disease* (4). The numbers, i.e., counts, of people in the respective states 1–4 are denoted by *S*, *C*, *D*_0_, and *D*_1_. Subjects may change their state along the arrows in Figure 1 as time *t* evolves.

### Simulation setup and estimation

To apply the theory in a realistic setting, we choose a test example from (7) motivated by the situation of type 2 diabetes in Germany. Diabetes is assumed to be irreversible. In the example, we have only one time scale and the time variable *t*, which is interpreted as age (7). For setting up the simulation, the age-specific incidence rate *i* is given by *i*(*t*) = max(0, *t*−30)/2,000 and the mortality rates *m*_0_ and *m*_1_ are assumed to be of Gompertz-type with *m*_0_(*t*) = exp(−10.7 + 0.1 *t*) and *m*_1_(*t*) = exp(−10 + 0.1 *t*). The simulation starts with a population of *N* = 10,000 study participants, for whom the changes of the states in the extended IDM as in the right part of Figure 1 are simulated by a micro-simulation (8). In short, for each subject possible transition times between the states in the IDM are randomly drawn by inverse transform sampling. Then, we mimic a sequence of eleven cross-sectional studies at time points *t*_1_ = 0, *t*_2_ = 10, …, *t*_11_ = 100. Each subject is randomly allocated to exactly one of the eleven cross-sections *t*_k_, where the vector *X*(*t*_k_) = (*S*(*t*_k_), *C*(*t*_k_), *D*_0_(*t*_k_), *D*_1_(*t*_k_)) of count data, is determined for *k* = 1,…, 11.

We assume that the functional form of the rates is known (incidence rate and Gompertz mortality rates) and the aim of our simulation study is to estimate the numeric parameters underlying the rates. This means, in statistical terms we have a six-dimensional parametric model [Definition 2.1 (9)] with parameters ϑ = (ϑ_1_, ϑ_2_, ϑ_3_, ϑ_4_, ϑ_5_, ϑ_6_), which we want to estimate from the simulated ACS data. The associated rates read as *i*(*t*) = ϑ_2_ × max(0, *t*−ϑ_1_), *m*_0_(*t*) = exp(ϑ_3_ + *t* × ϑ_4_) and *m*_1_(*t*) = exp(ϑ_5_ + *t* × ϑ_6_). The parameter ϑ_1_ is the age of onset of diabetes, which for type 2 diabetes in Germany is at the age of about 30 years. The parameter ϑ_2_ is the slope of the age course of the incidence rate. As a coarse approximation of the age-specific incidence rate in Germany, we choose an incidence rate that increases linearly with age starting at age 30. The remaining parameters ϑ_3_ to ϑ_6_ describe the two Gompertz mortality rates. The true parameter vector ϑ^(true)^ underlying the simulation is ϑ^(true)^ = (ϑ_1_, ϑ_2_, ϑ_3_, ϑ_4_, ϑ_5_, ϑ_6_) = (30, 1/2000, −10.7, 1/10, −10, 1/10). We will use the findings of the seminal work of (10) to derive a system of DEs that describe the extended IDM. The system of DEs will be the ground for two estimation methods for ϑ from the ACS data: the first is based on a least squares fit, the second is a maximum likelihood approach. The estimated ϑ for both methods will be compared to the true ϑ^(true)^ in terms of the absolute relative error.

The source code of the simulation and both estimation approaches in the free statistical programming language R (The R Foundation for Statistical Computing) is available from the open access public repository Zenodo under DOI 10.5281/zenodo.16789935 (11).

## Results

### Extended IDM and differential equations

We will use the extended IDM as in the right part of Figure 1. Let *S*(*t*), *C*(*t*), *D*_0_(*t*), and *D*_1_(*t*) denote the number of subjects in the respective state at time *t*. The non-negative transition rates may depend on time *t* and the fractions of people in the four states (numbered 1–4 in Figure 1) are denoted by *p*_j_ = *p*_j_(*t*), *j* = 1, 2, 3, 4 according to the numbers of the states. For example, at some time t* the fraction *p*_3_(t*) denotes the fraction of people, who are deceased without disease at t*. The fractions *p*_1_, *p*_2_, *p*_3_, and *p*_4_ always add up to 100%: *p*_1_(*t*) + *p*_2_(*t*) + *p*_3_(*t*) + *p*_4_(*t*) = 1 for all *t*. Furthermore, let *N*(*t*) = *S*(*t*) + *C*(*t*) + *D*_0_(*t*) + *D*_1_(*t*) and for brevity define the integer valued, four dimensional vector *X*(*t*): = (*S*(*t*), *C*(*t*), *D*_0_(*t*), *D*_1_(*t*)).

The work of Jahnke & Huisinga (10) from the context of state models proves that *X*(*t*) follows a multinomial distribution where the parameter *p* = *p*(*t*) of the multinomial distribution is the solution of the following system of ordinary differential equations (ODEs):

(1) $$\frac{d}{dt}p(t)\;=\;p^{\prime }(t)=\;A(t)\;p(t)$$

with initial condition *p*(0) = *p*_0_. The matrix *A*(*t*) in Equation (1) is given by

$$A(t)\;=\;\left[\begin{array}{cccc} -i(t)\;-m_{0}(t) & 0 & 0 & 0\\ i(t) & -m_{1}(t) & 0 & 0\\ m_{0}(t) & 0 & 0 & 0\\ 0 & m_{1}(t) & 0 & 0 \end{array}\right]$$

Using *p*(*t*) = (*p*_1_(*t*), *p*_2_(*t*), *p*_3_(*t*), *p*_4_(*t*)) and omitting the time dependency, we obtain the following linear system of ODEs:

(2a) $$p_{1}^{\prime }=-(i+m_{0})p_{1}$$

(2b) $$p_{2}^{\prime }\;=ip_{1}-\;m_{1}p_{2}$$

(2c) $$p_{3}^{\prime }\;=m_{0}p_{1}$$

(2d) $$p_{4}^{\prime }\;=m_{1}p_{2}.$$

Note that *p*(*t*) ∈ (0,1)^4^ is four-dimensional, but *p*_1_(*t*), *p*_2_(*t*), *p*_3_(*t*), *p*_4_(*t*) ∈ (0,1) are scalars.

We will use the four-dimensional system as in Equation (1) or the four equivalent scalar Equations (2a–d) to estimate the parameter ϑ underlying the rates *i*, *m*_0_, and *m*_1_ from the four-dimensional count vector *X*(*t*) = (*S*(*t*), *C*(*t*), *D*_0_(*t*), *D*_1_(*t*)).

### Simulation

After running the micro-simulation detailed in (8) with the rates *i*, *m*_0_ and *m*_1_ parameterized with ϑ^(true)^ as described above, we randomly assign the *N* = 10,000 simulated individuals to exactly one of the cross-sections at *t*_k_ = 0, …, 100. Table 1 shows the resulting number of subjects in each state at each of the eleven cross-sections. For example, at cross-section *t*_k_ = 60 a total of *N*_7_ = 899 were counted, with 677, 143, 55, and 24 in the states 1–4 (see the numbers in Figure 1), respectively. The total number *N*_k_ of participants at each of the eleven cross-sections ranges from 877 to 959. As the sum over all *S*(*t*_k_) equals 6,784, we see that the huge majority of sampled individuals are in health state 1, *Non-diseased*.

**Table 1:** Aggregated current status data at each of the eleven cross-sections.

*t*_k_	0	10	20	30	40	50	60	70	80	90	100	∑
*S*(*t*_k_)	906	932	958	878	845	794	677	499	233	59	3	6,784
*C*(*t*_k_)	0	0	0	0	22	82	143	221	116	20	1	605
*D*_0_(*t*_k_)	0	0	1	3	10	33	55	153	288	445	488	1,476
*D*_1_(*t*_k_)	0	0	0	0	0	5	24	67	249	380	410	1,135
*N*_k_	906	932	959	881	877	914	899	940	886	904	902	10,000

**Figure 2:**
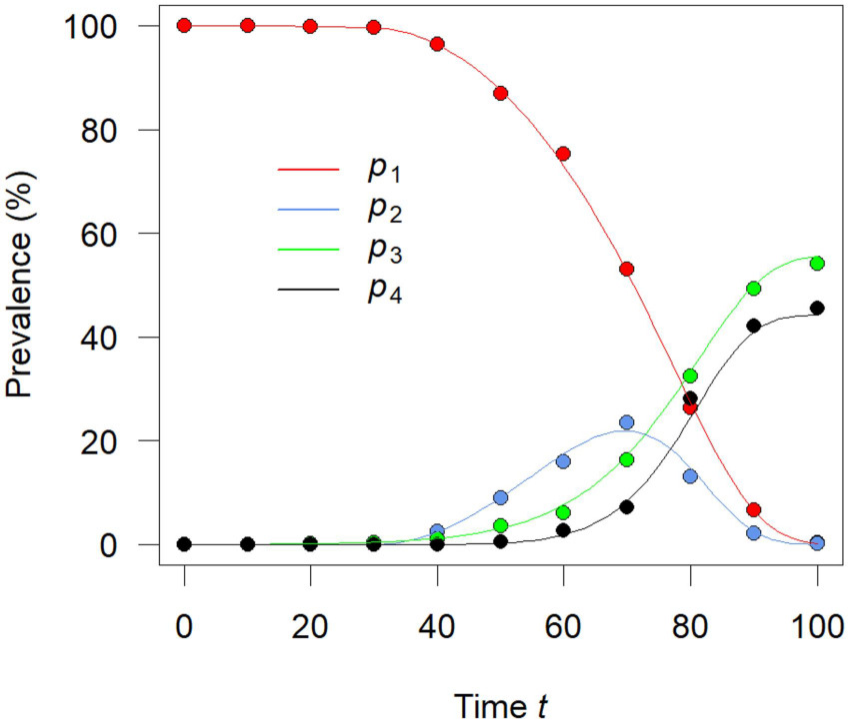
The four components of *p*^(obs)^ from the ACS data in Table 1 are shown as filled dots. For comparison, the associated components of the solution *p* of Equation (1) are shown as lines. The colors refer to the components, for example, green presents the third of the four components.

The resulting fractions *p*^(obs)^ are shown in Figure 2 as filled dots. For comparison, the associated components of the solution *p* of the ODE system (1) with the true parameters ϑ^(true)^ for *i*, *m*_0_ and *m*_1_ are shown as lines. Deviations of the dots from the solution show the random character of the ACS data [which come from a multinomial distribution (10)].

We use the system given in Eqs. (2a–d) to determine the parameters ϑ in the rates *i*, *m*_0_ and *m*_1_ from ACS data as shown in Table 1 and graphically depicted as dots in Figure 2.

### Least squares and maximum likelihood estimation

For a given parameter vector ϑ, we can solve Equations (2a–d) and obtain the solution *p*(*t*; ϑ). Then for the first estimation method, we may calculate the squared residual || *p*(; ϑ)−*p*^(obs)^ ||^2^ of the difference between *p*(; ϑ) and the observed fractions *p*^(obs)^ in the ACS data. The norm || · ||^2^ sums over all observed points in time *t*_k_, *k* = 1, …, *K*; for example in Table 1 from *t*_1_ = 0 to *t*_11_ = 100 in units of 10. The optimal ϑ* in the sense of least squares is given by

(3) $$\vartheta^{*}\;=\;\arg\;\min\;||p(;\vartheta )\;-p^{\left(\mathrm{obs}\right)}|{|}_{.}^{2}$$

In other words, ϑ* minimizes the squared difference between the modelled solution *p*(*t*; ϑ) of system Equations (2a–d) and the observed fractions *p*^(obs)^ ∈ (0,1)^4^; hence, ϑ* is a least squares estimator [Definition 3.1 in (9)].

In case that study participants at the *K* cross-sections are all independent, the log-likelihood function ℓ(ϑ) of the multinomial distribution can be used to estimate the unknown parameter ϑ = (ϑ_1_, ϑ_2_, ϑ_3_, ϑ_4_, ϑ_5_, ϑ_6_). Under independence we obtain

(4) $$\ell (\vartheta )\;=\;\overset{K}{\underset{k=1}{\sum }}\left[log(n_{k}!)\;+\;\overset{4}{\underset{\;j=1}{\sum }}\{x_{k,j}\;log(\;p_{j}(t_{k};\;\vartheta ))\;-\;log(x_{k,j}!)\}\right]$$

which can be maximized with respect to ϑ. This provides a second estimation method, a maximum likelihood estimator [Definition 4.3 in (9)]. In Equation (4) *x*_k,j_ denotes the number of subjects in state *j* at time *t*_k_, for *j* = 1, 2, 3, 4 and *k* = 1, …, *K*.

To sum up, for estimating ϑ we can use two different estimation methods: the least-squares minimization as in Equation (3) and the maximum likelihood estimation as in Equation (4). In both estimation approaches, we need to calculate the solution *p*(*t*; ϑ) for a given ϑ, which will be done by the classical Runge-Kutta method of fourth order [rk4 in the R package deSolve (12)]. The optimization method optim of the statistical software R provides the least squares estimates and the maximum likelihood estimates according to Equations (3, 4), respectively. Table 2 shows the results for both estimation methods based on the data in Table 1 together with the absolute relative error.

**Table 2:** Least squares and maximum likelihood estimates for the parameter ϑ with absolute relative errors (as percentages in brackets).

Parameter	True value ϑ^(true)^	Estimates ϑ* with absolute relative errors (%)	
		Least squares	Maximum likelihood
ϑ_1_	30	30.9 (3.3%)	30.3 (1.0%)
ϑ_2_ (per 10,000)	5	5.35 (7.1%)	5.16 (3.3%)
ϑ_3_	−10.7	−11.10 (3.8%)	−10.82 (1.1%)
ϑ_4_	0.1	0.105 (5.5%)	0.101 (1.1%)
ϑ_5_	−10	−10.0 (0.0%)	−9.77 (2.3%)
ϑ_6_	0.1	0.101 (1.5%)	0.098 (2.0%)

It is visible from Table 2 that both estimation methods provide estimates with an absolute relative error below 10%. It seems that the maximum likelihood estimates are slightly superior to the least squares estimates, because the absolute relative errors of the maximum likelihood method are consistently below 4%.

## Discussion

In this article, we have sketched how aggregated current status (ACS) data from the extended illness-death model (IDM) can be utilized to obtain information about the transition rates, the incidence (*i*) and the mortality rates (*m*_0_ and *m*_1_). IDM. We showed the feasibility of two novel estimation methods based on a system of ordinary differential equations (ODE): a least squares estimator and a maximum likelihood estimator. The least squares estimator uses the prevalence proportions *p* = (*p*_1_, *p*_2_, *p*_3_, *p*_4_), while the maximum likelihood estimator requires count data *X* = (*S*, *C*, *D*_0_, *D*_1_) (as in Table 1). The slight superiority of the maximum likelihood estimates compared to the least squares estimates in terms of absolute relative errors can be seen in the light of statistical efficiency. In short, the maximum likelihood estimation is the standard for asymptotic efficiency because it fully utilizes the distributional assumption of the data [see Theorem 6.2.2 in (13)]. The least squares estimator does not require a full distribution but therefore sacrifices efficiency. In case of the maximum likelihood estimation, 95% confidence bounds can be obtained with standard arguments from asymptotic statistics via inversion of the Fisher information matrix [Section 4.3 from (14)]. Unfortunately, the least squares estimator does not allow such an easy derivation of secondary order statistics. The least squares estimator requires further considerations for inferential measures, for example multidimensional resampling (15). However, secondary order statistics are beyond the scope of this feasibility study.

We think that the novel methods will be helpful in settings where follow-up data are not available. To estimate transition rates, usually cohort studies are run. ACS data, however, allude to a study design, where following-up of study participants is not necessary. Compared to simple prevalence data π: = *p*_2_/(*p*_1_ + *p*_2_), which is frequently reported in epidemiological studies, for the novel method described here, the number or percentage of deceased persons is necessary. These data about fatalities may be obtained from official residents’ registries (in case they exist). In case only the simple prevalence π is reported, we cannot estimate all rates of the IDM. Inserting Eqs. (2a,b) yields the ODE πʹ = (1−π)[*i*−π (*m*_1_−*m*_0_)], which compared to the four dimensional system (1) has the advantage of being scalar (making computations easier), but can only be used to estimate one rate from π (and πʹ) given the other two rates.

The possibility of using one study for estimating all three rates (incidence *i*, moralities *m*_0_ and *m*_1_) has an advantage for considering stochastic dependencies between the prevalence and the three rates. When two (or more) data sources are used to estimate measures of the IDM like e.g., in (2), usually these dependencies cannot be accounted for, because figures of covariance between prevalence and rates are not provided among the different data sources. If only one data source is used, it is possible to consider dependencies e.g., in multidimensional resampling (15) or bootstrapping (16).

The question arises, where ACS data play a role in practical applications. ACS data has the advantage that in many cases individual study subjects are non-identifiable, because data are aggregated and need not be reported on the individual level. This can be important for data protection reasons—especially if the health state *Ill* refers to particularly sensible diseases like sexually transmitted diseases. As an example for an application of ACS data, one might think of people with permanent need for long-term care in elderly population. In Germany, the mortality rate ratio (MRR) for these people is unknown. Follow-up studies are difficult because elderly people can be difficult to contact, withdrawal of consent is frequent and transport to examination centers imposes more problems than in a younger study population. Studies, which we think are suitable for application of the proposed method, are for example the Survey of Health, Ageing and Retirement in Europe (SHARE) and the Health and Retirement Study (HRS). Both studies provide large samples in an older population and collect comprehensive information about chronic conditions and mortality. Aggregated count data similar to Table 1 can be extracted after taking the survey weighting scheme into account. However, the practical aspects with weighting and secondary order statistics necessary for an appropriate analysis of these studies is beyond the scope of this feasibility study.

Usually when a study participant dies, the subject leaves the survey (“drop out”) and data is used only up to that point in time. In the proposed method, this is different: data can enter the analysis many years later after the fatality occurred. To illustrate this, we provide details of the most extreme case of the simulation described above. Study subject 3,512 (of the *N* = 10,000) dies young without contracting the disease at age 19.2 years. The data of subject 3,512 enter the ACS data of Table 1 more than 80 years later at the cross-section at *t*_k_ = 100. The subject is one of the subjects at the cell *D*_0_(100) = 488 in Table 1 above, which means that the statistical information of this young fatality is used no earlier than 80 years after death. The fact that information about the state (death) can be used for a data point more than 80 years later, requires that the health state does not change in the meantime (which is obvious in case of death). In case of incorrect assignments to health states, the estimates may become biased (misclassification). An example may be if a subject is counted towards *D*_0_ although the subject contracted the disease (and should hence be counted toward *D*_1_). This situation has been called misclassification of disease state at death (17).

The question arises how the method described here can be generalized to the case, when more than one time scale is involved. Our micro-simulation can be considered as a birth cohort, which implies that one time variable (here *t*) is sufficient to capture all relevant temporal changes. In epidemiological practice, frequently two times scales, calendar time and age, are involved, see for example (18). In this case, the ODE system in Equation (1) will become a partial differential equation (PDE) using the multidimensional chain rule [as described in (19)]. Since the PDE can be reduced to an ODE similar to Equation (1) by the method of characteristics (20), the techniques for treating two time scales are very similar to the methods described in this manuscript.

To sum up, we could show that the extended IDM can be used to obtain insights into all three transition rates. Two methods for estimating the parameters have been developed, one method is based on a least squares minimization and the other is based on maximum likelihood optimization. Applicability has been demonstrated in a simulation study motivated by diabetes in Germany. We find a good agreement between both estimation methods and the input used to set up the simulations.

## Data Availability

The datasets presented in this study can be found in online repositories. The names of the repository/repositories and accession number(s) can be found in the article/Supplementary Material.
